# Supplementary material for: Protein-Gene Orthology in Baculoviridae: An Exhaustive Analysis to Redefine the Ancestrally Common Coding Sequences
Source: Viruses. 2023 Apr 29;15(5):1091. doi: 10.3390/v15051091 (PMC10223672; doi:10.3390/v15051091)
Supplement: Supplementary file 1 [file viruses-15-01091-s001.zip › Supplementary material_Figures.pdf]

viruses

MDPI

SUPPLEMENTARY MATERIAL (Figures)

This section contains the supplementary **Figures S1**, **S2, S3, S4, S5 and S6**.

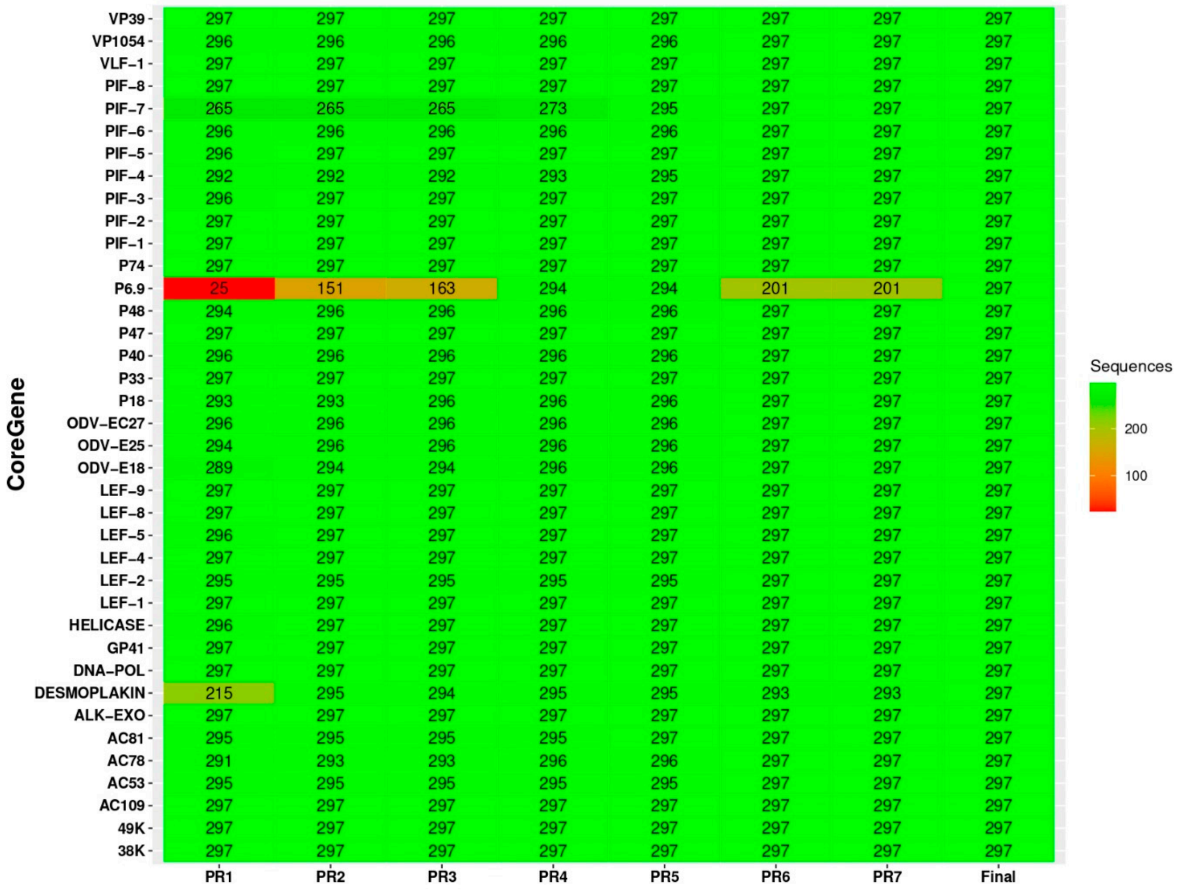

Figure S1. **Core gene results from the Ortholog Bioinformatic Pipeline**. Heat map showing the number of genomes (total 297) that are incorporated at each stage of the OBP for each of the 38 core genes accepted for baculoviruses. The name of core genes are indicated on the Y-axis while the OPB stages are indicated on the X-axis. PR: partial results; Final: final result (at the end of OBP execution). The color scale is referenced to the right of the table, approaching red when orthologous groups have few representatives of the 297 baculoviral genomes considered, and approaching green when homologous sequences are found in all genomes.

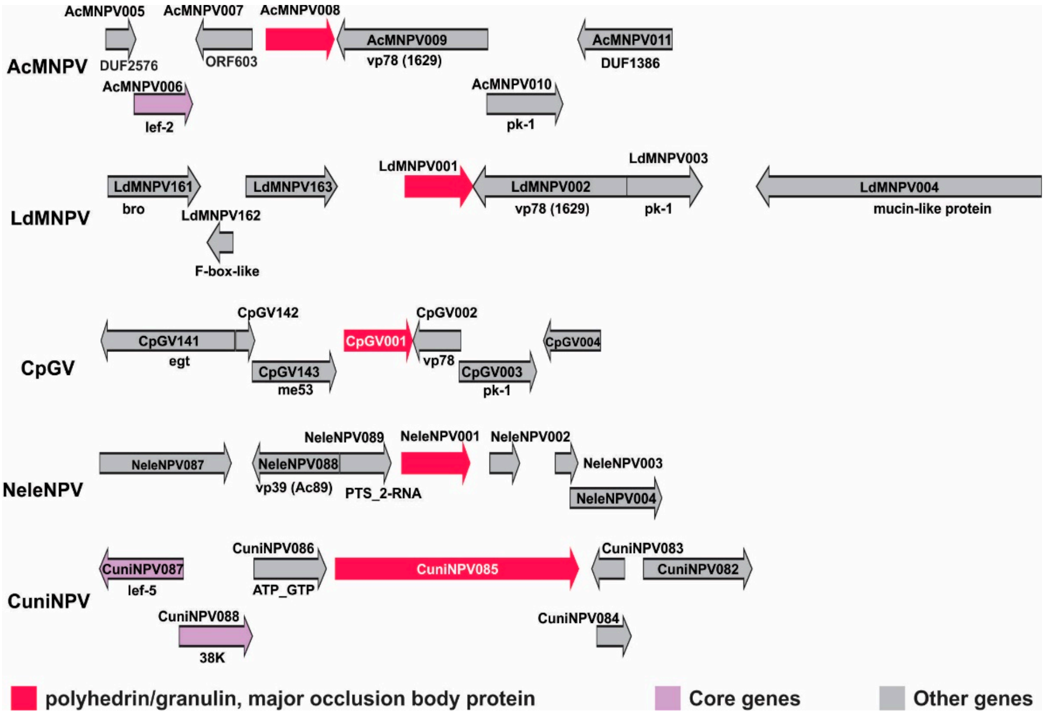

Figure S2. ***Major occlusion body protein* (*polyhedrin, granulin, CUN085*) gene synteny**. The illustration shows the genomic organization of the *major occlusion body protein* gene-containing regions in baculoviral prototypes (AcMNPV for *Alphabaculovirus* Group I; LdMNPV for *Alphabaculovirus* Group II; CpGV for *Betabaculovirus*; CuniNPV for *Deltabaculovirus*; NeleNPV for *Gammabaculovirus*). The names of the genes are shown according to the ORF number of each considered genome. The most accepted names for some of these genes are also indicated.

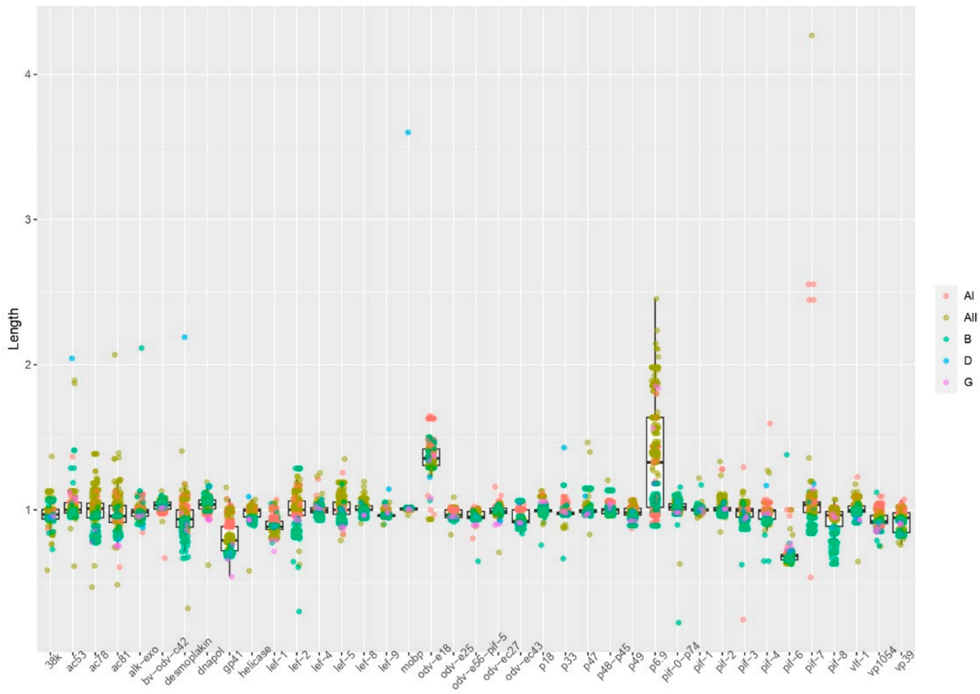

Figure S3. Protein length of baculovirus core genes. Box plot showing the length distribution of each of the proteins encoded by the 38 accepted core genes plus the group represented by polyhedrin/granulin/CUN085 (indicated as mobp by "major occlusion body protein"). To facilitate their comparison, the length of each AcMNPV protein was considered to have a value of 1, and this reference was used for the orthologs of the other 296 baculoviral genomes considered in this study. A1: alphabaculoviruses of Group I; A2: alphabaculoviruses of Group I; B: betabaculoviruses; D: deltabaculovirus; G: gammabaculoviruses.

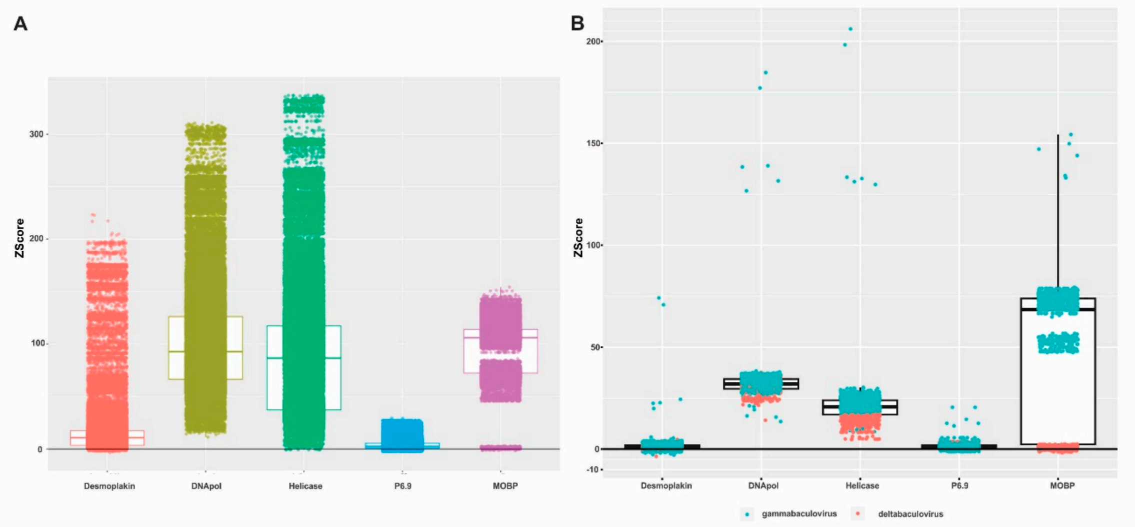

Figure S4. Z-Score analysis. Box plots showing the Z-score distribution for 4 accepted core proteins [Desmoplakin (Ac66), DNA polymerase (Ac65), DNA Helicase (Ac95), and P6.9 (Ac100)] and the newly proposed it (MOBP). Z scores for all proteins were calculated and are shown in panel **A**; for a better interpretation, in panel **B** only those for gamma- and deltabaculoviruses are shown. The line in zero is only a reference to show that Desmoplakin, DNA Helicase, and P6.9 have outliers below zero.

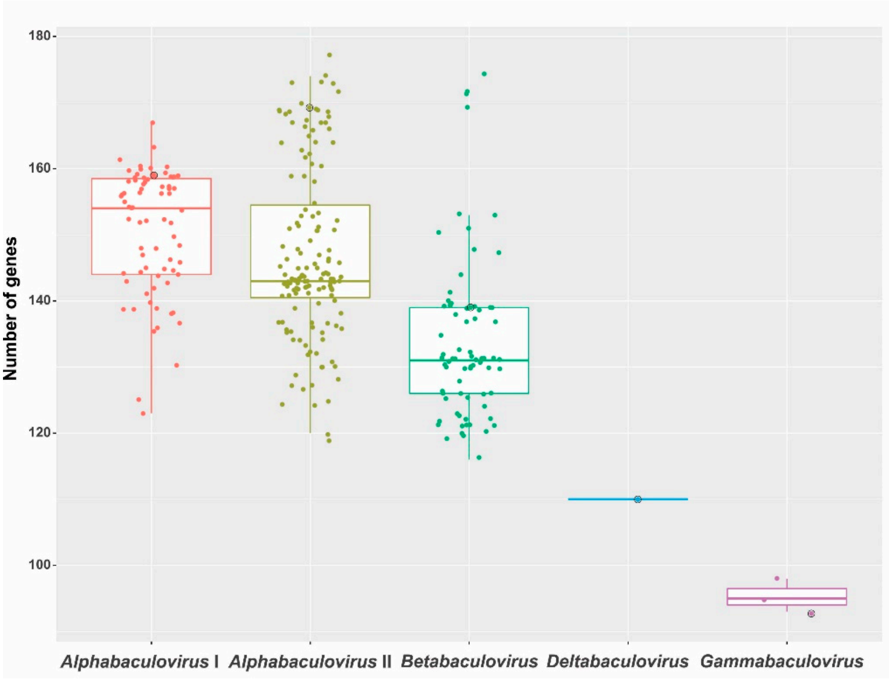

Figure S5. Content of protein-coding genes in baculoviral taxa. Box plot of the 297 baculoviral genomes according to their number of protein genes (ordinate axis) and considering taxa (abscissa axis). Each dot represents the datum for each genome. Only orthologous and unique genes detected in each genome are considered in the number of genes. Potentially paralogous copies are not counted. The value linked to each of the baculovirus prototypes (AcMNPV for *Alphabaculovirus* Group I; LdMNPV for *Alphabaculovirus* Group II; CpGV for *Betabaculovirus*; CuniNPV for *Deltabaculovirus*; NeleNPV for *Gammabaculovirus*) is indicated with a black circle that surrounds the dot.

A.

B.

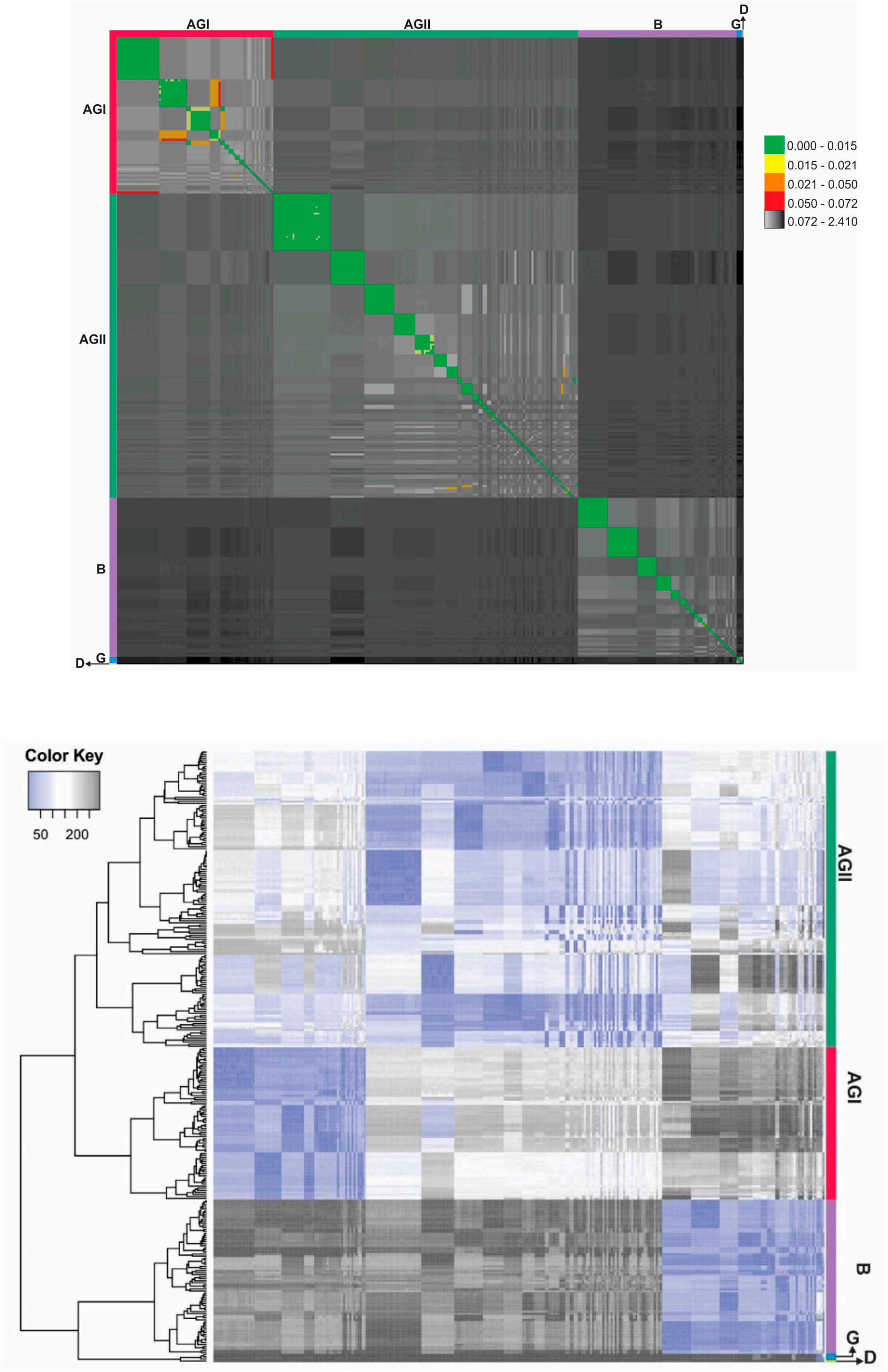

Figure S6. **Organization of baculoviruses according to K2P values.** Distances based on K2P data from 38 accepted core genes of baculoviruses were used to group the 297 members considered in this study. **A**. Representation of the K2P values in a heatmap using previously established color scales [16]. The baculoviral genera are indicated on the axes. A1: alphabaculoviruses of Group I; A2: alphabaculoviruses of Group I; B: betabaculoviruses; D: deltabaculovirus; G: gammabaculoviruses. **B**. Clustered heat map of K2P distance based on baculovirus core genes. The illustration was made using the Heatmap2 program from Galaxy server (https://usegalaxy.org). AG1: alphabaculoviruses of Group I; AG2: alphabaculoviruses of Group I; B: betabaculoviruses; D: deltabaculovirus; G: gammabaculoviruses.

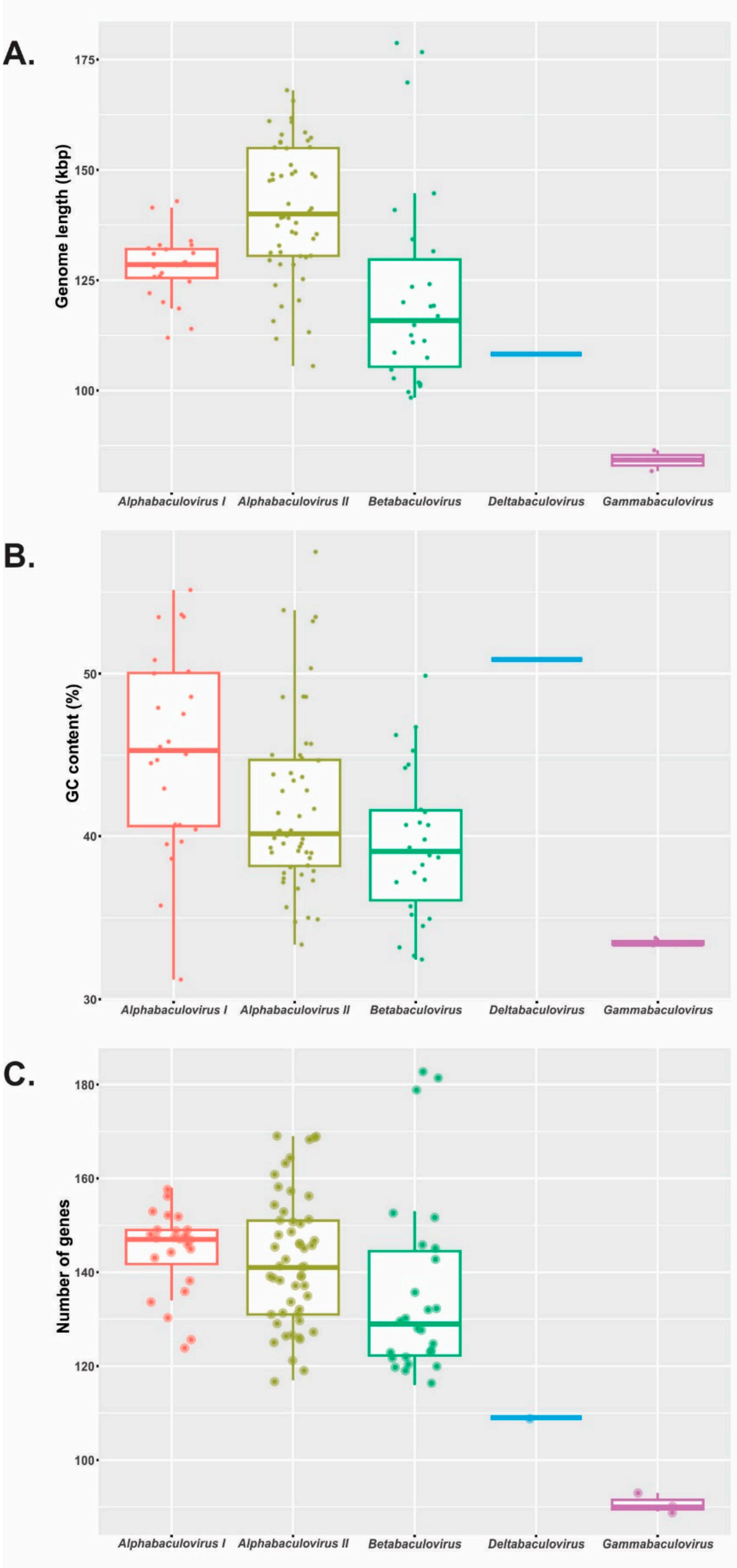

Figure S7. **Length, GC and gene content.** Equivalent analyses to those shown in Figures 2 and S5 were performed, but only one genome per baculoviral species was considered (a total of 107 baculoviruses). **A**. Genome length. **B**. GC content. **C**. Number of protein genes.
