# Supplementary material for: Protein-Gene Orthology in Baculoviridae: An Exhaustive Analysis to Redefine the Ancestrally Common Coding Sequences
Source: Viruses. 2023 Apr 29;15(5):1091. doi: 10.3390/v15051091 (PMC10223672; doi:10.3390/v15051091)
Supplement: Supplementary file 1 [file viruses-15-01091-s001.zip › Supplementary material_Tables.pdf]

viruses

MDPI

SUPPLEMENTARY MATERIAL (Tables)

This section contains supplementary **Tables S1, S2, S3, S4, S5, S6 and S7**.

Table S1. Baculoviruses and other invertebrate viruses used.

Alphabaculovirus Group I			
**Denomination**	**Strain/Isolate/Genotype**	**Acronym**	**GB Accession**
Antheraea pernyi MNPV	Isolate L2	AnpeMNPV-L2	EF207986
Antheraea pernyi NPV	Isolate Z	AnpeNPV-Z	NC_008035.3
Antheraea pernyi NPV	Strain Liaoning	AnpeNPV-Li	LC194889
Antheraea pernyi NPV	Liaoning	Anpe-CN	LC375540
Antheraea proylei NPV	Strain Manipur	AnprNPV-Ma	LC375539
Antheraea proylei NPV	Strain Manipur/ isolate AnprNPV-TS	AnprNPV-TS	KY979487
Antheraea proylei NPV	strain TkhulenIBD	AnprNPV-TK	MH797002
Antheraea yamamai NPV	Strain Nagano	AnyaNPV-Na	LC375537
Anticarsia gemmatalis MNPV	Isolate 2D	AgMNPV	NC_008520.2
Anticarsia gemmatalis MNPV	Isolate Ag-01	AgMNPV-01	MG746625
Anticarsia gemmatalis MNPV	Isolate Ag-16	AgMNPV-16	MG746626
Anticarsia gemmatalis MNPV	Isolate 26	AgMNPV-26	KR815455
Anticarsia gemmatalis MNPV	Isolate 27	AgMNPV-27	KR815456
Anticarsia gemmatalis MNPV	Isolate 28	AgMNPV-28	KR815457
Anticarsia gemmatalis MNPV	Isolate 29	AgMNPV-29	KR815458
Anticarsia gemmatalis MNPV	Isolate 30	AgMNPV-30	KR815459
Anticarsia gemmatalis MNPV	Isolate 31	AgMNPV-31	KR815460
Anticarsia gemmatalis MNPV	Isolate 32	AgMNPV-32	KR815461
Anticarsia gemmatalis MNPV	Isolate 33	AgMNPV-33	KR815462
Anticarsia gemmatalis MNPV	Isolate 34	AgMNPV-34	KR815463
Anticarsia gemmatalis MNPV	Isolate 35	AgMNPV-35	KR815464
Anticarsia gemmatalis MNPV	Isolate 36	AgMNPV-36	KR815465
Anticarsia gemmatalis MNPV	Isolate 37	AgMNPV-37	NC_031761
Anticarsia gemmatalis MNPV	Isolate 38	AgMNPV-38	KR815467
Anticarsia gemmatalis MNPV	Isolate 39	AgMNPV-39	KR815468
Anticarsia gemmatalis MNPV	Isolate 40	AgMNPV-40	KR815469
Anticarsia gemmatalis MNPV	Isolate 42	AgMNPV-42	KR815470
Anticarsia gemmatalis MNPV	Isolate 43	AgMNPV-43	KR815471
Autographa californica MNPV	Clone C6	AcMNPV	NC_001623
Autographa californica MNPV	Strain E2	AcMNPV-E2	KM667940
Autographa californica MNPV	Isolate WP10	AcMNPV-WP10	KM609482
Bombyx mandarina NPV	Isolate S1	BomaNPV-S1	NC_012672
Bombyx mandarina NPV	Isolate S2	BomaNPV-S2	JQ071499
Bombyx mori NPV	Isolate T3	BmNPV	NC_001962
Bombyx mori NPV	strain Cubic	BmNPV-Cub	JQ991009
Bombyx mori NPV	strain Guangxi	BmNPV-Gua	JQ991011
Bombyx mori NPV	strain India	BmNPV-Ind	JQ991010
Bombyx mori NPV	strain Zhejiang	BmNPV-Zhe	JQ991008
The table with Group I alphabaculoviruses continues the next page			
**Denomination**	**Strain/Isolate/Genotype**	**Acronym**	**GB Accession**
Bombyx mori NPV	isolate C1	BmNPV-C1	KF306215
Bombyx mori NPV	isolate C2	BmNPV-C2	KF306216

Bombyx mori NPV	isolate C6	BmNPV-C6	KF306217
Bombyx mori NPV	strain Brazilian	BmNPV-Bra	KJ186100
Bombyx mori NPV	strain La	BmNPV-La	LC500465
Bombyx mori NPV	isolate Baoshan	BmNPV-Bao	MT501299
Catopsilia pomona	isolate 416	CapoNPV	NC_030240
Choristoneura fumiferana MNPV	--	CfMNPV	NC_004778.3
Choristoneura fumiferana Def MNPV	--	CfDEFMNPV	NC_005137.2
Choristoneura murinana NPV	Strain Darmstadt	ChmuNPV	NC_023177
Choristoneura occidentalis NPV	Isolate BC1	ChocNPV	NC_021925
Choristoneura rosaceana NPV	Isolate NB1	ChroNPV	NC_021924
Choristoneura diversana NPV	Isolate ChdiNPV-Hokkaido	ChdiNPV	LC516821
Condylorrhiza vestigialis MNPV	--	CoveMNPV	NC_026430
Cyclophragma undans NPV	Isolate Whiov	CyunNPV-Wh	NC_055467
Dasychira pudibunda NPV	Isolate ML1	DapuNPV	KP747440
Dendrolimus kikuchii NPV	strain YN	DekiNPV	JX193905
Dione juno NPV	isolate Araguari-MG	Araguari-MG	MK558262
Epiphyas postvittana NPV	--	EppoNPV	NC_003083
Hyphantria cunea NPV	--	HycuNPV	NC_007767
Hyphantria cunea NPV	isolate BJ	HycuNPV-BJ	OL686894
Hyphantria cunea NPV	isolate HB	HycuNPV-HB	OL686893
Lonomia obliqua MNPV	isolate SP/2000	LoobMNPV	NC_043520
Maruca vitrata NPV	--	MaviNPV	NC_008725
Orgyia pseudotsugata MNPV	--	OpMNPV	NC_001875.2
Orgyia pseudotsugata SNPV	isolate MEM	OpSNPV-MEM	MZ127289
Oxyplax ochracea NPV	isolate 435	OxocNPV-435	NC_043529
Philosamia cynthia ricini NPV	--	PhcyNPV	JX404026
Plutella xylostella MNPV	Isolate CL3	PlxyMNPV	NC_008349
Rachiplusia nu MNPV	isolate VPN54	RanuMNPV	MK419956
Samia cynthia NPV	Isolate Nagano	SacyNPV-Na	LC375538
Samia ricini NPV	Isolate Guangxi	SariNPV-Gu	LC375541
Samia ricini NPV	Isolate Mondulkiri	SariNPV-Mo	LC375542
Samia ricini NPV	Isolate Son La	SariNPV-SL	LC375543
Spilosoma obliqua NPV	Isolate IIPR	SpobNPV-IIPR	KY550224
Thysanoplusia orichalcea NPV	Isolate P2	ThorNPV-P2	NC_019945

Group II alphabaculoviruses continue the next page.

Alphabaculovirus Group II			
**Denomination**	**Strain/Isolate/Genotype**	**Acronym**	**GB Accession**
Adoxophyes honmai NPV	--	AdhoNPV	NC_004690
Adoxophyes orana NPV	--	AdorNPV	NC_011423
Agrotis ipsilon MNPV	--	AgipMNPV	NC_011345
Agrotis segetum NPV	--	AgseNPV	NC_007921
Agrotis segetum NPV	B isolate English	AgseNPV-Beng	NC_025960
Apocheima cinerarium NPV	--	ApciNPV	NC_018504
Buzura suppressaria NPV	Isolate Hubei	BusuNPV	NC_023442
Buzura suppressaria NPV	Isolate Guangxi	BusuNPV-G	KM986882
Buzura suppressaria NPV	isolate BisuNPV-CNPSo-25	BisuNPV-CNPSo-25	MT561366
Chrysodeixis chalcites NPV	--	ChchNPV	NC_007151
Chrysodeixis chalcites NPV	genotype ChchSNPV-TF1-C	ChchNPV-TF1C	JX560539
Chrysodeixis chalcites NPV	genotype ChchSNPV-TF1-H	ChchNPV- TF1H	JX560542
Chrysodeixis chalcites NPV	genotype ChchSNPV-TF1-B	ChchSNPV-TF1B	JX560540

Chrysodeixis chalcites NPV	genotype ChchSNPV-TF1-G	ChchSNPV-TF1G	JX560541
Chrysodeixis chalcites NPV	genotype ChchSNPV-TF1-A	ChchSNPV-TF1A	JX535500
Chrysodeixis includens NPV	isolate ChinNPV-1	ChinNPV-1	MK746083
Chrysodeixis includens NPV	isolate ChinNPV-MG.A	ChinNPV-MGA	MN542939
Chrysodeixis includens NPV	isolate ChinNPV-MG.B	ChinNPV-MGB	MN542938
Chrysodeixis includens NPV	isolate ChinNPV-MT.A	ChinNPV-MTA	MN689112
Chrysodeixis includens NPV	isolate ChinNPV-MT.B	ChinNPV-MTB	MN689113
Chrysodeixis includens NPV	isolate ChinNPV-MT.C	ChinNPV-MTC	MN689114
Chrysodeixis includens NPV	isolate ChinNPV-MT.D	ChinNPV-MTD	MN689115
Chrysodeixis includens NPV	isolate ChinNPV-MT.E	ChinNPV-MTE	MN689116
Clanis bilineata NPV	Isolate DZ1	ClbiNPV	NC_008293
Cryptophlebia peltastica	Isolate SA	CrpeNPV	NC_055500
Ectropis obliqua NPV	Strain A1	EcobNPV	NC_008586
Ectropis obliqua NPV	Strain Unioasis 1	EcobNPV-U1	KC960018
Ectropis obliqua NPV	strain QF4	EcoNPV-QF4	MZ394738
Erannis ankeraria	isolate wlcb	EranNPV	OK091173
Euproctis digramma NPV	strain 424	ArdiNPV	MN233792
Euproctis pseudoconspersa NPV	--	EupsNPV	NC_012639
Gynaephora ruoergensis NPV	--	GyruNPV	MW173388
Helicoverpa armigera MNPV	--	HaMNPV	NC_011615
Helicoverpa armigera NPV	Isolate Australia	HaNPV-Aus	JN584482
Helicoverpa armigera NPV	Strain C1	HaNPV-C1	NC_003094.2
Helicoverpa armigera NPV	Strain G4	HaNPV-G4	NC_002654.2
Helicoverpa armigera SNPV	Strain NNg1	HaSNPV	NC_011354
Helicoverpa armigera SNPV	isolate H25EA1	HaSNPV-H25EA1	KJ922128
Helicoverpa armigera SNPV	Strain AC53	HaSNPV-AC53	NC_024688
Helicoverpa armigera NPV	Strain SP1A	HaNPV-SP1A	KJ701032
Helicoverpa armigera NPV	Strain SP1B	HaNPV-SP1B	KJ701033
Helicoverpa armigera NPV	Strain L1	HaNPV-L1	KT013224
Helicoverpa armigera NPV	Strain LB1	HaNPV-LB1	KJ701029
Helicoverpa armigera NPV	Strain LB3	HaNPV-LB3	KJ701030
Helicoverpa armigera NPV	Strain LB6	HaNPV-LB6	KJ701031
The table with Group II alphabaculoviruses continues the next page			
**Denomination**	**Strain/Isolate/Genotype**	**Acronym**	**GB Accession**
Helicoverpa armigera NPV	Strain AC53T4.1	HaSNPV-T4-1	KU738902
Helicoverpa armigera NPV	Strain AC53T4.2	HaSNPV-T4-2	KU738903
Helicoverpa armigera NPV	Strain AC53C1	HaSNPV-C1	KU738896
Helicoverpa armigera NPV	Strain AC53C3	HaSNPV-C3	KU738897
Helicoverpa armigera NPV	Strain AC53C5	HaSNPV-C5	KU738898
Helicoverpa armigera NPV	Strain AC53C6	HaSNPV-C6	KU738899
Helicoverpa armigera NPV	Strain AC53C9	HaSNPV-C9	KU738900
Helicoverpa armigera NPV	isolate HearNPV-TR	HearNPV-TR	MK507817
Helicoverpa armigera NPV	strain WV104		MT810812
Helicoverpa assulta NPV	isolate HasNPV-DJ0031	HasNPV-DJ0031	MG569706
Helicoverpa SNPV	Strain AC53T2	HaSNPV-T2	KU738901
Helicoverpa SNPV	Strain AC53T5	HaSNPV-T5	KU738904
Helicoverpa zea NPV	--	HezeNPV	NC_003349
Helicoverpa zea SNPV	Strain HS-18	HezeSNPV-HS18	KJ004000
Helicoverpa zea SNPV	isolate Br/South	HezeSNPV-Br/South	KM596835
Hemileuca sp NPV	--	HespNPV	NC_021923
Hyposidra talaca NPV	--	HytaNPV	NC_055453.1
Lambdina fiscellaria NPV	isolate GR15	LafiNPV-GR15	NC_026922

Leucania separata NPV	Strain AH1	LeseNPV	NC_008348
Lymantria dispar MNPV	--	LdMNPV	NC_001973
Lymantria dispar MNPV	Isolate LdMNPV-27	LdMNPV-27	KP027546.2
Lymantria dispar MNPV	Strain 3029	LdMNPV-3029	KM386655
Lymantria dispar MNPV	isolate 2161	LdMNPV-2161	KF695050.2
Lymantria dispar MNPV	isolate BNP	LdMNPV-BNP	KU377538
Lymantria dispar MNPV	isolate RR01	LdMNPV-RR01	KX618634
Lymantria dispar MNPV	isolate Aba624	LdMNPV-Aba624	KT626572
Lymantria dispar MNPV	strain 3041	LdMNPV-3041	KT626571
Lymantria dispar MNPV	strain 3054	LdMNPV-3054	KT626570
Lymantria dispar MNPV	isolate 45/0	LdMNPV-45/0	KU862282
Lymantria dispar MNPV	strain H2	LdMNPV-H2	MK264918
Lymantria dispar MNPV	strain J2	LdMNPV-J2	MK089451
Lymantria dispar MNPV	isolate HrB	LdMNPV-HrB	MT782112
Lymantria dispar MNPV	isolate HrB-NJSS	LdMNPV-HrB-NJSS	MT782113
Lymantria dispar MNPV	isolate LDMN_Nsk06	LdMNPV-NsK06	MK411291
Lymantria dispar MNPV	isolate LDMN_Nsk07	LdMNPV-NsK07	MK411292
Lymantria dispar MNPV	strain NSh-07	LdMNPV-NSh07	MN661137
Lymantria xylina MNPV	isolate LyxyMNPV-5	LyxyMNPV	NC_013953
Malacosoma neustria NPV	isolate ManeNPV-T2	ManeNPV-T2	NC_040606
Mamestra brassicae MNPV	Isolate Chb1	MabrMNPV-Chb1	JX138237.2
Mamestra brassicae MNPV	isolate CTa	MabrMNPV- CTa	KJ871680
Mamestra brassicae MNPV	Isolate K1	MabrMNPV-K1	NC_023681
Mamestra configurata NPV	Strain A90-2	McNPV-90-2	NC_003529
Mamestra configurata NPV	Strain A90-4	McNPV-90-4	AF539999
Mamestra configurata NPV	Strain B	McNPV-B	NC_004117
Mamestra configurata NPV	isolate 94-2	McNPV-94	MN320360
Mamestra configurata NPV	isolate Ls	McNPV-Ls	MN395659
Mamestra configurata NPV	isolate SK256	McNPV-SK256	MN337871
Mamestra configurata NPV	isolate SK156	McNPV-SK156	MK252969
Mamestra configurata NPV	isolate AB260	McNPV-AB260	MK409385
The table with Group II alphabaculoviruses continues the next page			
**Denomination**	**Strain/Isolate/Genotype**	**Acronym**	**GB Accession**
Mythimna unipuncta NPV	strain #7	MyunNPV-7	NC_043530
Mythimna unipuncta NPV	strain KY310	MyunNPV-KY310	MH124167
Neophasia NPV	isolate 11	NespNPV	MK293724
Olene mendosa NPV	isolate 435	OlmeNPV	MZ766431
Operophtera brumata NPV	Isolate MA	OpbrNPV-MA	NC_040621
Orgyia leucostigma NPV	Isolate CFS77	OrleNPV	NC_010276
Peridroma sp NPV	isolate GR-167	PespNPV	NC_024625
Perigonia lusca SNPV	--	PeluSNPV	NC_027923
Pseudoplusia includens SNPV	IE	PsinSNPV-IE	NC_026268
Pseudoplusia includens SNPV	IA	PsinSNPV-IA	KU669289
Pseudoplusia includens SNPV	IB	PsinSNPV-IB	KU669290
Pseudoplusia includens SNPV	IC	PsinSNPV-IC	KU669291
Pseudoplusia includens SNPV	ID	PsinSNPV-ID	KU669292
Pseudoplusia includens SNPV	IF	PsinSNPV-IF	KU669293
Pseudoplusia includens SNPV	IG	PsinSNPV-IG	KU669294
Spodoptera cosmioides NPV	isolate VPN72	SpcoNPV	MK419955
Spodoptera eridania NPV	isolate 251	SperNPV-251	NC_055502
Spodoptera eridania NPV	isolate CNPSo-165	SperNPV-CNPSo-165	MT040195
Spodoptera exempta NPV	strain 244.1	SpexNPV	NC_055455

Spodoptera exigua MNPV	--	SeMNPV	NC_002169
Spodoptera exigua MNPV	isolate HT-SeG25	SeMNPV-HT-SeG25	HG425347.2
Spodoptera exigua MNPV	isolate HT-SeG24	SeMNPV-HT-SeG24	HG425346
Spodoptera exigua MNPV	isolate VT-SeAl1	SeMNPV-VT-SeAl1	HG425343
Spodoptera exigua MNPV	isolate HT-SeSP2A	SeMNPV-HT-SeSP2A	HG425349.2
Spodoptera exigua MNPV	isolate HT-SeG26	SeMNPV-HT-SeG26	HG425348
Spodoptera exigua MNPV	isolate VT-SeAl2	SeMNPV-VT-SeAl2	HG425344
Spodoptera exigua MNPV	isolate VT-SeOx4	SeMNPV-VT-SeOx4	HG425345
Spodoptera exigua MNPV	isolate SeMNPV-QD	SeMNPV-QD	MH370144
Spodoptera exigua MNPV	isolate WV103	SeMNPV-WV103	MN481987
Spodoptera frugiperda MNPV	Isolate 3AP2	SfMNPV-3AP2	NC_009011.2
Spodoptera frugiperda MNPV	Isolate Nicaraguan	SfMNPV-Nic	HM595733
Spodoptera frugiperda MNPV	Isolate Nicaraguan DefG	SfMNPV-NicG	JF899325
Spodoptera frugiperda MNPV	Strain 19	SfMNPV-19	EU258200
Spodoptera frugiperda MNPV	Isolate Colombian	SfMNPV-Col	KF891883
Spodoptera frugiperda MNPV	isolate ARG-M	SfMNPV-ARGM	MW162628
Spodoptera frugiperda MNPV	isolate IIBBL BCIPV 1197	SfMNPV-1197	MK503925
Spodoptera frugiperda MNPV	isolate IIBBL BCIPV 281	SfMNPV-281	MK503923
Spodoptera frugiperda MNPV	isolate IIBBL BCIPV 459	SfMNPV-459	MK503924
Spodoptera frugiperda MNPV	isolate KA1	SfMNPV-AKA1	MZ292981
Spodoptera littoralis NPV	Isolate AN1956	SpliNPV-AN1956	NC_038369
Spodoptera littoralis NPV	isolate SpliNPV-Tun2	SpliNPV-Tun2	MG958660
Spodoptera litura II MNPV	--	SpltMNPV-II	NC_011616
Spodoptera litura MNPV	Strain G2	SpltMNPV-G2	NC_003102
Spodoptera litura NPV	Strain WV108	SpltMNPV-WV108	MZ290131
Sucra jujuba NPV	isolate 473	SujuNPV-473	KJ676450
Trabala vishnou gigantina NPV	strain wuqi	TrviNPV	MW645368
Troides aeacus NPV	isolate wt	TraeNPV	MH077961
Trichoplusia ni SNPV	--	TnSNPV	NC_007383
The table with Group II alphabaculoviruses continues the next page			
**Denomination**	**Strain/Isolate/Genotype**	**Acronym**	**GB Accession**
Trichoplusia ni SNPV	isolate LBIV-4	TnSNPV	MH577296
Urbanus proteus NPV	isolate Southern Brazil	UrprNPV	NC_029997.2

Betabaculoviruses continue the next **Table S1** section.

Betabaculovirus			
**Denomination**	**Strain/Isolate/Genotype**	**Acronym**	**GB Accession**
Adoxophyes orana GV	--	AdorGV	NC_005038
Adoxophyes orana GV	Strain Miyazaki	AdorGV-Miy	KM226332
Agrotis segetum GV	--	AgseGV	AY522332.4
Agrotis segetum GV	strain L1	AgseGV-L1	KC994902
Agrotis segetum GV	strain DA	AgseGV-DA	NC_039213
Choristoneura occidentalis GV	--	ChocGV	NC_008168
Clostera anachoreta GV	Isolate HBHN	ClanGV	NC_015398
Clostera anastomosis GV	Strain Henan	CalGV-H	NC_022646
Clostera anastomosis GV	Strain ClasGV-B	ClasGV-B	NC_038371
Cnaphalocrocis medinalis GV	Strain Enping	CnmeGV-E	NC_029304.2
Cnapahlocrocis medinalis GV	--	CnmeGV	KP658210
Cryptophlebia leucotreta GV	--	CrleGV	NC_005068
Cydia pomonella GV	--	CpGV	NC_002816
Cydia pomonella GV	isolate CpGV-E2	CpGV-E2	KM217577

Cydia pomonella GV	isolate CpGV-S	CpGV-S	KM217573
Cydia pomonella GV	isolate CpGV-I12	CpGV-I12	KM217576
Cydia pomonella GV	isolate CpGV-M	CpGV-M	KM217575
Cydia pomonella GV	isolate CpGV-I07	CpGV-I07	KM217574
Cydia pomonella GV	isolate CpGV-ALE	CpGV-ALE	MN696165
Cydia pomonella GV	isolate CpGV-JQ	CpGV-JQ	MN696166
Cydia pomonella GV	isolate CpGV-KS1	CpGV-KS1	MN696167
Cydia pomonella GV	isolate CpGV-KS2	CpGV-KS2	MN696168
Cydia pomonella GV	isolate CpGV-SA	CpGV-SA	MN075941
Cydia pomonella GV	isolate CpGV-WW	CpGV-WW	MN696169
Cydia pomonella GV	isolate CpGV-ZY	CpGV-ZY	MN696171
Cydia pomonella GV	isolate CpGV-ZY2	CpGV-ZY2	MN696170
Diatraea saccharalis GV	isolate DisaGV-Parana-2009	DisaGV-P09	NC_028491
Epinotia aporema GV	--	EpapGV	NC_018875
Erinnyis ello ello GV	Strain BrS86	ErelGV	NC_025257
Erinnyis ello GV	ErelGV_Isolate 00	ErelGV-00	KX859082
Erinnyis ello GV	ErelGV_Isolate 94	ErelGV-94	KX859079
Erinnyis ello GV	ErelGV_Isolate 98	ErelGV-98	KX859080
Erinnyis ello GV	ErelGV_Isolate 99	ErelGV-99	KX859081
Erinnyis ello GV	ErelGV_Isolate AC	ErelGV-AC	KX859083
Erinnyis ello GV	ErelGV_Isolate PA	ErelGV-PA	KX859084
Helicoverpa armigera GV	--	HearGV	NC_010240
Hyphantria cunea GV	isolate Hc1	HycuGV-Hc1	MH923363

The table with betabaculoviruses continues the next page

Denomination	Strain/Isolate/Genotype	Acronym	GB Accession
Matsumuraeses phaseoli GV	strain IOZ01	MaphGV	MT844067
Mocis latipes GV	isolate Southern Brazil	MolaGV	NC_029996
Mythimna unipuncta GV	isolate MyunGV#8	MyunGV	NC_033780.2
Phthorimaea operculella GV	--	PhopGV	NC_004062
Phthorimaea operculella GV	isolate SA	PhopGV-SA	KU666536
Phthorimaea operculella GV	isolate PhopGV-CR3.1	PhopGV-CR3.1	MK033565
Phthorimaea operculella GV	isolate PhopGV-CR5.1	PhopGV-CR5.1	MK033566
Phthorimaea operculella GV	isolate PhopGV-CR1.1	PhopGV-CR1.1	MK033567
Phthorimaea operculella GV	isolate PhopGV-CR1.2	PhopGV-CR1.2	MK033568
Phthorimaea operculella GV	isolate PhopGV-CR2.1	PhopGV-CR2.1	MK033569
Phthorimaea operculella GV	isolate PhopGV-IT1.1	PhopGV-IT1.1	MK033570
Phthorimaea operculella GV	isolate PhopGV-LS1.1	PhopGV-LS1.1	MK033571
Phthorimaea operculella GV	isolate PhopGV-LS1.2	PhopGV-LS1.2	MK033572
Phthorimaea operculella GV	isolate PhopGV-LS2.1	PhopGV-LS2.1	MK033573
Phthorimaea operculella GV	isolate PhopGV-LS3.1	PhopGV-LS3.1	MK033574
Phthorimaea operculella GV	isolate PhopGV-R	PhopGV-R	MK033575
Phthorimaea operculella GV	isolate PhopGV-Ym.1	PhopGV-Ym.1	MK033576
Pseudaletia unipuncta GV	Strain Hawaiin	PsunGV	NC_013772
Pieris rapae GV	Isolate Wuhan	PiraGV	NC_013797
Pieris rapae GV	Strain E3	PiraGV-E3	GU111736
Pieris rapae GV	South Korea	PiraGV-SK	JX968491
Pieris rapae GV	strain WV1010	PiraGV-WV1010	MW306663
Plodia interpunctella GV	isolate Cambridge	PlinGV	NC_032255
Plutella xylostella GV	--	PxGV	NC_002593
Plutella xylostella GV	isolate SA	PlxyGV-SA	KU666537
Plutella xylostella GV	isolate C	PlxyGV-C	KU529791
Plutella xylostella GV	isolate K	PlxyGV-K	KU529792

Plutella xylostella GV	isolate M	PlxyGV-M	KU529793
Plutella xylostella GV	isolate T	PlxyGV-T	KU529794
Plutella xylostella GV	isolate W	PlxyGV-W	MN099284
Plutella xylostella GV	isolate B	PlxyGV-B	MN099285
Plutella xylostella GV	isolate NW	PlxyGV-NW	MN099286
Spodoptera frugiperda GV	isolate VG008	SpfrGV	NC_026511
Spodoptera frugiperda GV	isolate ARG	SpfrGV-ARG	MH170055
Spodoptera litura GV	Strain K1	SpltGV	NC_009503
Trichplusia ni GV	isolate LBIV-12	TrniGV-LBIV12	KU752557
Xestia c nigrum GV	--	XecnGV	NC_002331

Deltabaculovirus, gammabaculoviruses and other invertebrate viruses continue the next **Table S1** section.

Deltabaculovirus			
**Denomination**	**Strain/Isolate/Genotype**	**Acronym**	**GB Accession**
Culex nigripalpus NPV	Florida1997	CuniNPV	NC_003084
Gammabaculovirus			
**Denomination**	**Strain/Isolate/Genotype**	**Acronym**	**GB Accession**
Neodiprion lecontei NPV	--	NeleNPV	NC_005906
Neodiprion sertifer NPV	--	NeseNPV	NC_005905
Neodiprion abietis NPV	--	NeabNPV	DQ317692

Invertebrate viruses (non-baculoviruses) used.

**Denomination**	**Strain/Isolate**	**Acronym**	**GB Accession**
Nudiviridae			
Gryllus bimaculatus nudivirus	--	GrBNV	NC_009240.1
Helicoverpa zea nudivirus 2	--	HzNV-2	NC_004156.2
Hytrosaviridae			
Musca domestica salivary gland hypertrophy virus	--	MdSGHV	NC_010671.1
Glossina pallidipes salivary gland hypertrophy virus	--	GpSGHV	NC_010356.1
Nimaviridae			
White spot syndrome virus	CN01	WSS-CN01	NC_003225.3
White spot syndrome virus	MEX2008	WSS-MEX2008	KU216744

Table S2. Ortholog groups for baculovirus protein genes.

This table is presented in a separate excel file due to its complexity. It shows the orthologous gene groups after the application of OBP. The first row indicates the genomes according to their accession number in GenBank (see **Table S1** for the virus denomination), and the first column indicates the genes using their most frequent name. The cells in the intersection indicate the presence (Y) or non-detection (N) of the corresponding gene. If the gene is detected, its genomic position is indicated.

Table S3. Orthologs without candidate gene in deltabaculovirus.

Baculovirus Protein*	Synteny conserved?	Conserved protein motif detected among homologous sequences from all baculoviruses	Protein from CuniNPV	% identity**
**Ac106**	No	YKDFNRRLLLIDF	CUN053	10.5-20.2
**DNA-binding (Ac25)**	No	YEIGLRVHGGPCPFIFWDFVTIKGCKGDFGEFF	not detected	--
**Ac75**	No	--	not detected	--
**Lef-11**	No	CLTRSEIYALTREAINHLKHTNDTEGVCAHHFD	CUN076	6.8-27.7
**Chtb1 (Ac145)**	No	--	not detected	--
**Ac76**	No	--	not detected	--
**P11 (Ac108)**	No	--	not detected	--
**Calyx/Pep/Pp34 (Ac131)**	No	LWVSADEVLQLLRLP	CUN103	7.3-19.6
**IAP (Ac27)**	No	--	not detected	--

* Homologs detected in all members of alpha-, beta-, and gammabaculoviruses. ** Range containing the minimum and maximum identity values obtained from comparing the corresponding CuniNPV protein with respect to the putative homologous ones belonging to the remaining 296 baculoviruses studied.

Table S4. Gene orthology among some members of invertebrate virus families.

	Invertebrate virus					
**Baculovirus core gene**	GrBNV	HzNV-2	GpSGHV	MdSGHV	WSS-CN01	WSS-MEX2008
**38k**	Y	Y	N	N	N	N
**ac53**	N	N	N	N	N	N
**ac78**	Y	N	Y	N	N	N
**ac81**	Y	Y	Y	Y	N	N
**alk-exo**	N	N	N	N	N	N
**bv-odv-c42**	N	N	N	N	N	N
**desmoplakin**	N	N	Y	N	Y	Y
**DNA polymerase**	N	N	Y	Y	Y	Y
**DNA helicase**	Y	Y	Y	Y	Y	Y
**lef-1**	N	N	N	N	N	N
**lef-2**	N	N	N	N	N	N
**lef-4**	N	N	N	N	N	N
**lef-5**	Y	Y	Y	N	N	N
**lef-8.**	N	N	N	N	N	N
**lef-9**	N	N	N	N	N	N
**odv-e18**	Y	N	Y	Y	N	N
**odv-e25**	N	N	N	N	N	N
**odv-ec27**	N	N	N	N	N	N
**odv-ec43**	N	N	N	N	N	N
**p18**	N	N	N	N	N	N
**p33**	N	N	N	N	N	N
**p47**	N	N	N	N	N	N
**p48-p45**	N	N	N	N	N	N
**p49**	N	N	N	N	N	N
**p6.9**	N	N	N	N	N	N
**pif-0-p74**	Y	Y	Y	Y	Y	Y
**pif-1**	Y	Y	Y	Y	N	N
**pif-2**	Y	Y	Y	Y	Y	Y
**pif-3**	Y	Y	Y	Y	Y	Y
**pif-4**	Y	Y	N	N	N	N
**pif-5**	Y	Y	Y	Y	N	N
**pif-6**	N	N	N	N	N	N

**pif-7**	Y	Y	Y	N	Y	Y
**pif-8**	N	N	N	N	N	N
**vlf-1**	N	N	N	N	N	N
**vp1054**	N	N	N	N	N	N
**vp39**	N	N	N	N	N	N

In this study, only the 38 core genes accepted in *Baculoviridae* were considered. Detection of a gene homologous to the corresponding baculoviral gene is indicated by "Y"; non-detection of a gene homologous to the corresponding baculoviral gene is indicated by "N". Genes detected in all studied genomes are highlighted with gray shading. The complete virus names are indicated in **Table S1**.

Table S5. Baculovirus unique genes.

This table is presented in a separate excel file due to its complexity. It shows the unique genes (sequences that did not exhibit evident homology with those of other baculoviruses after the application of OBP). The first row indicates the genomes according to their accession number in GenBank (see **Table S1** for the virus denomination), and the first column indicates the putative genes with the names indicated by their authors. The corresponding genomic positions are indicated.

Table S6. Baculovirus paralogous genes.

Paralogous group	Major protein domain detected (IntrerPro)
94k	-
bro	BRO family, N-terminal domain n (PF02498)
dbp	ssDNA binding protein (PF04786)
helicase	P-loop containing nucleoside triphosphate hydrolases (SSF52540)
iap-2	Inhibitor of apoptosis (SSF57924)
odv-e66	Baculovirus E66 occlusion-derived virus envelope protein (PF04850)
orf57	-
orf66	Dual specificity phosphatase, catalytic domain (PF00782)
p26	Nucleopolyhedrovirus p26 protein (PF04766)
rr2a	Ribonucleotide reductase, small chain (PF00268)
hypothetical-protein-1	Per os infectivity (PF05092)
hypothetical-protein-10	Autographa californica nuclear polyhedrosis virus (AcMNPV), Orf17 (PF06856)
hypothetical-protein-11	-
hypothetical-protein-12	Nucleic acid-binding proteins (SSF50249)
hypothetical-protein-13	Protein of unknown function (DUF2661) (PF10860)
hypothetical-protein-14	-
hypothetical-protein-15	Family of unknown function (DUF5863) (PF19181)
hypothetical-protein-16	-
hypothetical-protein-18	-
hypothetical-protein-19	Proliferating cell nuclear antigen signature (PS00293)
hypothetical-protein-2	Baculoviridae p74 conserved region (PF04583)
hypothetical-protein-20	Peptidase family M60 domain profile (PS51723)
hypothetical-protein-21	Nucleopolyhedrovirus P10 protein (PF05531)
hypothetical-protein-23	DNA photolyase (PF00875)
hypothetical-protein-24	DNA ligase/mRNA capping enzyme, catalytic domain (SSF56091)
hypothetical-protein-25	-
hypothetical-protein-26	Putative transposase DNA-binding domain (PF07282)
hypothetical-protein-27	-
hypothetical-protein-28	-
hypothetical-protein-29	-
The table continues the next page	
**Paralogous group**	**Major protein domain detected (IntrerPro)**

hypothetical-protein-3	Per os infectivity factor 3 (PF05006)
hypothetical-protein-30	-
hypothetical-protein-31	-
hypothetical-protein-36	-
hypothetical-protein-37	-
hypothetical-protein-38	-
hypothetical-protein-39	-
hypothetical-protein-4	Chitin binding Peritrophin-A domain (PF01607)
hypothetical-protein-40	-
hypothetical-protein-41	Zinc finger RING-type profile (PS50089)
hypothetical-protein-42	-
hypothetical-protein-43	-
hypothetical-protein-44	Virus envelope protein E26 (PF11050)
hypothetical-protein-45	L domain-like (SSF52058)
hypothetical-protein-46	-
hypothetical-protein-5	Baculovirus 19 kDa protein conserved region (PF04798)
hypothetical-protein-6	Baculovirus U-box/Ring-like domain (PF05883)
hypothetical-protein-7	(Trans)glycosidases (SSF51445)
hypothetical-protein-8	ChaB (PF06150)

The **Table S6** indicates homologous gene clusters where some genomes have more than 1 representative.

Table S7. Core genes among baculovirus genera.

	AcMNPV #ORF	Gene name and description	Previously reported genera*	Genera determined in this work*
Core genes for *Alpha-*, *Beta-*, and *Gammabaculovirus*	8	*polyhedrin granulin,* OB protein	A + B + G	A + B +G + D
	25	*dbp,* DNA binding protein	A + B + G	A + B + G
	37	*lef-11,* Required for expression of late genes	A + B + G	A + B + G**
	75	*ac75,* Required for BV production	A + B + G	A + B + G
	76	*ac76, Unknown function*	A + B + G	A + B + G
	106/107	*ac106/107,* Unknown function	A + B + G	A + B + G**
	108	*ac108,* P11 protein, associated with the PIF complex	A + B + G	A + B + G
	131	*pe/pp34,* Involved in morphogenesis of polyhedral envelope	A + B + G	A + B + G**
	145	*ac145,* OB protein, involved in oral infection *(Ac145 and 150 are predicted to encode a domain thought to bind to chitin)*	A + B + G	A + B + G
	27	*iap (-1, -2, -3)*	A + B + G	A + B + G
Core gene for *Alpha-*, *Beta-*, and *Deltabaculovirus*	23	*ac23*, *f-protein*, Pathogenicity factor in alphabaculoviruses of group I; fusion protein in alphabaculoviruses of group II, betabaculoviruses, and deltabaculoviruses	A + B + D	A + B + D
Core genes for *Alpha-*, and *Betabaculovirus*	10	*pk-1,* Protein kinase	A + B	A + B
	13	*ac13,* Unknown function	A + B	A + B
	28	*lef-6,* Involved in late and very late gene expression	A + B	A + B
	35	*ubiquitin,* Ubiquitin	A + B	A + B
	36	*pp31,* Late gene expression factor	A + B	A + B
	38	*ac 38, nudix* ADP-ribose pyrophosphatase	A + B	A + B
	61	*fp/25k,* Structural protein of BVs and ODVs	A + B + G	A + B

	67	*lef-3,* ssDNA binding protein	A + B	A + B
	82	*tlp,* Unknown function	A + B	A + B
	102	*p12,* Involved in nuclear localization of G-actin	A + B	A + B
	129	*p24,* ODV protein	A + B	A + B
	139	*me53,* DNA synthesis regulator, required for efficient BV production	A + B	A + B
	141	*Exon0,* Egress of nucleocapsids from the nucleus to the cytoplasm, interaction with btubulin and microtubules	A + B	A + B
	146	*ac146,* Unknown function	A + B	A + B
	147	*ie-1,* Immediate-early transactivator	A + B	A + B

*A = *Alphabaculovirus*; B = *Betabaculovirus*; D = *Deltabaculovirus*; G = *Gammabaculovirus.* ** More information for CuniNPV ORF candidates in **Table S3**.

Table S8. Distribution of baculoviral species based on K2P distances.

Genus	Group	Species	#Species	Genomes	ID GenBank	Denomination
Alpha	I	24	1	20	KR815455.1, KR815456.1, KR815457.1, KR815458.1, KR815459.1, KR815460.1, KR815461.1, KR815462.1, KR815463.1, KR815464.1, KR815465.1, KR815467.1, KR815468.1, KR815469.1, KR815470.1, KR815471.1, MG746625.1, MG746626.1, NC_008520.2, NC_031761.1	Anticarsia gemmatalis MNPV
			2	13	JQ071499.1, JQ991008.1, JQ991009.1, JQ991010.1, JQ991011.1, KF306215.1, KF306216.1, KF306217.1, KJ186100.1, LC500465.1, MT501299.1, NC_001962.1, NC_012672.1	Bombyx mandarina NPV (JQ071499.1, NC_012672.1), Bombyx mori NPV
			3	2	JX404026.1, LC375541.1	Philosamia cynthia ricini NPV (JX404026.1), Samia ricini NPV (LC375541.1)
			4	9	EF207986.1, KY979487.1, LC194889.1, LC375537.1, LC375538.1, LC375539.1, LC375540.1, MH797002.1, NC_008035.3	Antheraea pernyi MNPV (EF207986.1, NC_008035.3), Antheraea proylei NPV (KY979487.1, LC194889.1LC375537.1, LC375539.1, LC375540.1, MH797002.1), Samia cynthia NPV (LC375538.1)
			5	5	KM609482.1, KM667940.1, NC_001623.1, NC_008349.1, MH077961.2	Autographa californica MNPV, Plutella xylostella MNPV (NC_008349.1), Troides aeacus NPV (MH077961.2)
			6	2	LC375542.1, LC375543.1	Samia ricini NPV
			7	2	KP747440.1, NC_001875.2	Dasychira pudibunda NPV (KP747440.1), Orgyia pseudotsugata MNPV (NC_001875.2)
			8	2	LC516821.1, NC_023177.1	Choristoneura diversana nucleopolyhedrovirus ChdiNPV, Choristoneura murinana (NC_023177.1)
			9	3	KY550224.1, NC_004778.3, NC_021925.1	Spilosoma obliqua NPV (KY550224.1), Choristoneura fumiferana MNPV (NC_004778.3), Choristoneura occidentalis NPV (NC_021925.1)
			10	2	OL686893.1, OL686894.1	Hyphantria cunea NPV

			11	1	JX193905.1	Dendrolimus kikuchii NPV
			12	1	MK558262.1	Dione juno NPV
			13	1	MK293724.1	Neophasia NPV
			14	1	NC_003083.1	Epiphyas postvittana NPV
			15	1	NC_008725.1	Maruca vitrata NPV
			16	1	NC_007767.1	Hyphantria cunea NPV
			17	1	NC_019945.1	Thysanoplusia orichalcea NPV
			18	1	NC_021924.1	Choristoneura rosaceana NPV
			19	1	NC_030240.1	Catopsilia pomona
			20	1	NC_026430.1	Condylorrhiza vestigialis MNPV
			21	1	NC_043520.1	Lonomia obliqua MNPV
			22	1	NC_043529.1	Oxyplax ochracea NPV
			23	1	NC_055467.1	Cyclophragma undans NPV
			24	1	NC_005137.2	Choristoneura fumiferana Def MNPV
	**II**	**52**	1	27	JN584482.1, KJ004000.1, KJ701029.1, KJ701030.1, KJ701031.1, KJ701032.1, KJ701033.1, KJ922128.1, KM596835.1, KT013224.1, KU738896.1, KU738897.1, KU738898.1, KU738899.1, KU738900.1, KU738901.1, KU738902.1, KU738903.1, KU738904.1, MG569706.1, MK507817.1, MT810812.1, NC_002654.2, NC_003094.2, NC_003349.1, NC_011354.1, NC_024688.1	Helicoverpa armigera NPV (JN584482.1, KJ701029.1, KJ701030.1, KJ701031.1, KJ701032.1, KJ701033.1, KT013224.1, KU738896.1, KU738897.1, KU738898.1, KU738899.1, KU738900.1, KU738902.1, KU738903.1, NC_003094.2), Helicoverpa zea SNPV (KJ004000.1, KM596835.1, MK507817.1, MT810812.1, NC_002654.2, NC_003349.1), Helicoverpa SNPV (KU738901.1. KU738904.1), Helicoverpa assulta NPV (MG569706.1), Helicoverpa armigera SNPV (NC_011354.1, NC_024688.1, KJ922128.1)
			2	16	KF695050.2, KM386655.1, KP027546.2, KT626570.1, KT626571.1, KT626572.1, KU862282.1, KX618634.1, MK089451.1, MK264918.1, MK411291.1, MK411292.1, MN661137.1, MT782112.1, MT782113.1, NC_001973.1	Lymantria dispar MNPV
			3	14	KU669289.1, KU669290.1, KU669291.1, KU669292.1, KU669293.1, KU669294.1, MN542938.1, MN542939.1, MN689112.1, MN689113.1, MN689114.1, MN689115.1, MN689116.1, NC_026268.1	Chrysodeixis includens (KU669289.1, KU669290.1, KU669291.1, KU669292.1, KU669293.1, KU669294.1, MN542938.1, MN542939.1, MN689112.1, MN689113.1, MN689114.1, MN689115.1, MN689116.1), Pseudoplusia includens SNPV (NC_026268.1)
			4	10	EU258200.1, HM595733.1, JF899325.1, KF891883.1, MK503923.1, MK503924.1, MK503925.1, MW162628.1, MZ292981.1, NC_009011.2	Spodoptera frugiperda MNPV
			5	9	HG425343.1, HG425344.1, HG425345.1, HG425346.1, HG425347.2, HG425348.1, HG425349.2, MN481987.1, NC_002169.1	Spodoptera exigua MNPV
			6	6	JX138237.2, KJ871680.1, MN337871.1, NC_004117.1, NC_011615.1, NC_023681.1	Mamestra brassicae MNPV (JX138237.2, KJ871680.1, NC_023681.1), Mamestra configurata NPV (MN337871.1, NC_004117.1), Helicoverpa armigera MNPV (NC_011615.1)
			7	5	AF539999.1, MK409385.1, MN320360.1, MN395659.1, NC_003529.1	Mamestra configurata NPV
			8	3	MZ394738.1, NC_008586.1, KC960018.1	Ectropis obliqua NPV
			9	5	JX535500.1, JX560539.1, JX560541.1, JX560542.1, NC_007151.1	Chrysodeixis chalcites NPV

			10	3	KM986882.1, MT561366.1, NC_023442.1	Buzura suppressaria NPV
			11	2	MG958660.1, NC_038369.1	Spodoptera littoralis NPV
			12	2	MH577296.1, NC_007383.1	Trichoplusia ni SNPV
			13	2	MZ290131.1, NC_003102.1	Spodoptera litura NPV (MZ290131.1), Spodoptera litura MNPV (NC_003102.1)
			14	2	MW173388.1, NC_012639.1	Gynaephora ruoergensis NPV, Euproctis pseudo-conspersa NPV (NC_012639.1)
			15	1	MZ127289.1	Orgyia pseudotsugata SNPV
			16	1	MH124167.1	Mythimna unipuncta NPV
			17	1	MH370144.1	Spodoptera exigua MNPV
			18	1	MK419956.1	Rachiplusia nu MNPV
			19	1	MK419955.1	Spodoptera cosmioides NPV
			20	1	MK746083.1	Chrysodeixis includens NPV
			21	1	MN233792.1	Euproctis digramma NPV
			22	1	MT040195.1	Spodoptera eridania NPV
			23	1	MW645368.1	Trabala vishnou gigantina NPV
			24	1	MZ766431.1	Olene mendosa NPV
			25	1	NC_004690.1	Adoxophyes honmai NPV
			26	1	NC_007921.1	Agrotis segetum NPV
			27	1	NC_008293.1	Clanis bilineata NPV
			28	1	NC_008348.1	Leucania separata NPV
			29	1	NC_010276.1	Orgyia leucostigma NPV
			30	1	NC_011345.1	Agrotis ipsilon MNPV
			31	1	NC_011423.1	Adoxophyes orana NPV
			32	1	NC_013953.1	Lymantria xylina MNPV
			33	1	NC_018504.1	Apocheima cinerarium NPV
			34	1	NC_021923.1	Hemileuca sp NPV
			35	1	NC_024625.1	Peridroma sp NPV
			36	1	NC_025960.1	Agrotis segetum NPV
			37	1	NC_026922.1	Lambdina fiscellaria NPV
			38	1	NC_027923.1	Perigonia lusca SNPV
			39	1	NC_029997.2	Urbanus proteus NPV
			40	1	NC_040606.1	Malacosoma neustria NPV
			41	1	NC_040621.1	Operophtera brumata NPV
			42	1	NC_043530.1	Mythimna unipuncta NPV
			43	1	NC_055455.1	Spodoptera exempta NPV
			44	1	NC_055500.1	Cryptophlebia peltastica
			45	1	OK091173.1	Erannis ankeraria
			46	1	NC_055453.1	Hyposidra talaca NPV
			47	1	JX560540.1	Chrysodeixis chalcites NPV
			48	1	MK252969.1	Mamestra configurata NPV
			49	1	NC_011616.1	Spodoptera litura II MNPV
			50	1	NC_055502.1	Spodoptera eridania NPV
			51	1	KJ676450.1	Sucra jujuba NPV
			52	1	KU377538.1	Lymantria dispar MNPV
Beta	**--**	**26**	1	14	KM217573.1, KM217574.1, KM217575.1, KM217576.1, KM217577.1, MN075941.1, MN696165.1, MN696166.1, MN696167.1, MN696168.1, MN696169.1, MN696170.1, MN696171.1, NC_002816.1	Cydia pomonella GV

			2	14	KU666536.1, MK033565.1, MK033566.1, MK033567.1, MK033568.1, MK033569.1, MK033570.1, MK033571.1, MK033572.1, MK033573.1, MK033574.1, MK033575.1, MK033576.1, NC_004062.1	Phthorimaea operculella GV
			3	9	KU529791.1, KU529792.1, KU529793.1, KU529794.1, KU666537.1, MN099284.1, MN099285.1, MN099286.1, NC_002593.1	Plutella xylostella GV
			4	7	KX859079.1, KX859080.1, KX859081.1, KX859082.1, KX859083.1, KX859084.1, NC_025257.1	Erinnyis ello GV (KX859079.1, KX859080.1, KX859081.1, KX859082.1, KX859083.1, KX859084.1), Erinnyis ello ello GV (NC_025257.1)
			5	4	GU111736.1, JX968491.1, MW306663.1, NC_013797.1	Pieris rapae GV
			6	3	KC994902.1, NC_005839.2, NC_039213.1	Agrotis segetum GV
			7	2	KM226332.1, NC_005038.1	Adoxophyes orana GV
			8	2	KP658210.1, NC_029304.2	Cnaphalocrocis medinalis GV
			9	2	MH170055.1, NC_026511.1	Spodoptera frugiperda GV
			10	2	KU752557.1, NC_013772.1	Trichplusia ni GV (KU752557.1), Pseudaletia unipuncta GV (NC_013772.1)
			11	1	NC_002331.1	Xestia c nigrum GV
			12	1	NC_010240.1	Helicoverpa armigera GV
			13	1	MH923363.1	Hyphantria cunea GV
			14	1	MT844067.1	Matsumuraeses phaseoli GV
			15	1	NC_005068.1	Cryptophlebia leucotreta GV
			16	1	NC_008168.1	Choristoneura occidentalis GV
			17	1	NC_009503.1	Spodoptera litura GV
			18	1	NC_015398.1	Clostera anachoreta GV
			19	1	NC_018875.1	Epinotia aporema GV
			20	1	NC_022646.1	Clostera anastomosis GV
			21	1	NC_028491.1	Diatraea saccharalis GV
			22	1	NC_029996.1	Mocis latipes GV
			23	1	NC_032255.1	Plodia interpunctella GV
			24	1	NC_033780.2	Mythimna unipuncta GV
			25	1	NC_038371.1	Clostera anastomosis GV
			26	1	SPORGV2022.1	Spodoptera ornithogalli GV
Gamma	**--**	**3**	1	1	DQ317692.1	Neodiprion abietis NPV
			2	1	NC_005905.1	Neodiprion sertifer NPV
			3	1	NC_005906.1	Neodiprion lecontei NPV
Delta	**--**	**1**	1	1	NC_003084.1	Culex nigripalpus NPV
